# A Digital Intervention Using Daily Financial Incentives to Increase Medication Adherence in Severe Mental Illness: Single-Arm Longitudinal Pilot Study

**DOI:** 10.2196/37184

**Published:** 2022-10-12

**Authors:** Daniel Guinart, Michael Sobolev, Bhagyashree Patil, Megan Walsh, John M Kane

**Affiliations:** 1 Department of Psychiatry The Zucker Hillside Hospital Glen Oaks, NY United States; 2 Institute of Behavioral Science The Feinstein Institutes for Medical Reseach Manhasset, NY United States; 3 Department of Psychiatry and Molecular Medicine Zucker School of Medicine at Hofstra/Northwell Hempstead, NY United States; 4 Institut de Neuropsiquiatria i Addiccions, Parc de Salut Mar Barcelona Spain; 5 Institut Hospital del Mar d'Investigacions Mèdiques Barcelona Spain; 6 Centro de Investigación Biomédica en Red de Salud Mental Barcelona Spain; 7 Cornell Tech Cornell University New York, NY United States

**Keywords:** antipsychotic, adherence, digital, mobile health, mHealth, financial incentives

## Abstract

**Background:**

Medication nonadherence is prevalent in severe mental illness and is associated with multiple negative outcomes. Mobile technology and financial incentives show promise to improve medication adherence; however, studies in mental health, especially with oral medications, are lacking.

**Objective:**

The aim of this paper is to assess the feasibility and effectiveness of offering financial incentives through a mobile app based on behavioral economics principles to improve medication adherence in severe mental illness.

**Methods:**

A 10-week, single-arm longitudinal pilot study was conducted. Patients earned rewards in the context of app-based adherence incentives. The reward was split into biweekly payments made in increments of US $15, minus any US $2 per day penalties for missed check-ins. Time-varying effect modeling was used to summarize the patients’ response during the study.

**Results:**

A total of 25 patients were enrolled in this pilot study, of which 72% (n=18) were female, and 48% (n=12) were of a White racial background. Median age was 24 (Q1-Q3: 20.5-30) years. Participants were more frequently diagnosed with schizophrenia and related disorders (n=9, 36%), followed by major depressive disorder (n=8, 32%). App engagement and medication adherence in the first 2 weeks were higher than in the last 8 weeks of the study. At study endpoint, app engagement remained high (n=24, Z=–3.17; *P*<.001), but medication adherence was not different from baseline (n=24, Z=–0.59; *P*=.28).

**Conclusions:**

Financial incentives were effectively delivered using an app and led to high engagement throughout the study and a significantly increased medication adherence for 2 weeks. Leveraging behavioral economics and mobile health technology can increase medication adherence in the short term.

**Trial Registration:**

ClinicalTrials.gov NCT04191876; https://clinicaltrials.gov/ct2/show/NCT04191876

## Introduction

Medication adherence is a challenge in all of medicine, as only an average of 50% of individuals affected by a chronic condition follow their care plan as prescribed [1]. In mental health, poor adherence is a significant public health challenge, fueled by chronicity, lack of insight, significant medication side effects, and other factors such as stigma and poor access to care [2-4]. Neuropsychiatric medication reduces the severity of serious mental illness and improves patient outcomes [5-9], but only for as long as the patient is adherent. Unfortunately, the rates of adherence to neuropsychiatric medication are far from optimal, which has been estimated to average 40%-50% for schizophrenia and bipolar disorder [10-13]. Similar rates are reported for major depressive disorder [14,15], with some studies reporting rates as low as 21% at 12 months, albeit with variations by drug type [16].

Medication nonadherence has been associated with increased risk of relapse, violence, and legal problems; increased risk of suicide attempts; use of emergency services; and poor social and occupational functioning [17-23]. Recently, financial reinforcement interventions based on behavioral economic principles have emerged as a potential tool to enhance medication adherence in severe mental illness [24,25]. A very recent study explored the use of financial incentives to increase oral antidepressant adherence [26], and 2 studies have focused on antipsychotic medication [27,28], both limited to long-acting injectables. To our knowledge, no study to date has examined the effects of financial incentives on adherence to neuropsychiatric treatments including oral antipsychotic medication.

For this project, we used an app that takes advantage of behavioral economics principles to increase adherence for patients with chronic diseases [29]. Mobile health technology can be designed to be persuasive and potentially increase medication adherence when coupled with incentives contingent on behavior [30,31]. The aim of this study was to assess the feasibility and effectiveness of offering financial incentives through an app to help improve adherence to oral medication in severe mental illness.

## Methods

### Ethics Approval

This study was carried out in accordance with the Declaration of Helsinki [32], and all participants provided written informed consent as approved by the local Institutional Review Board (IRB#190739).

### Study Design

A 10-week, single-arm longitudinal pilot study was conducted (NCT04191876). Included were English-speaking patients 18-80 years old owning a smartphone and receiving treatment with psychotropic medication, with suspected or confirmed poor oral medication adherence. Patients were recruited from inpatient and outpatient units from a semiurban tertiary care facility that draws a representative racial or ethnic and sociodemographic mixture of eligible patients.

After consent, patients were instructed to download the study app, assisted by a digital navigator when necessary. The app automatically prompts the participant to take their medication by generating a reminder at a preset time of day. Patients were instructed to take a photo of the medication in their hand, as prescribed by their doctor, and submit it through the app, which was considered as a check-in or engagement. Engagement was defined as the number of app check-ins. Additionally, all photo check-ins were manually reviewed and verified by the study personnel to ensure accuracy and estimate reliability. Adherence was calculated by dividing the number of pills collected by the app at every check-in by the total number of pills required to be taken and was monitored throughout the study. Baseline adherence in relation to the number of pills required to be taken was determined by subject self-reports at the time of enrollment, which were then confirmed on the health care system’s electronic medical records as well as administrative data from the Medicaid claims database when available. Medication changes occurring during the study period were taken into account, and the number of pills to be taken was adjusted accordingly.

Patients were not compensated for participation in this project. They earned rewards in the context of adherence incentives based on successful check-ins. The reward was split into 5 biweekly payouts made in increments of US $15, minus any US $2 per day penalties for missed check-ins, up to a maximum reward of US $75 per participant over the study period. This incentive design is based on the loss aversion strategy, which has shown to be more powerful than gain-framed incentives in daily health behaviors such as physical activity [33] and smoking cessation [34].

### Data Analysis

Measures of mean engagement and adherence were used to summarize patient’s response over the 10 weeks of the study using intercept-only time-varying effect modelling (TVEM) [35,36]. We selected intercept-only TVEM to summarize longitudinal trends with 95% confidence intervals. This approach uses a spline function to approximate the average change in engagement and adherence over time [37]. Wilcoxon signed-rank test were used to compare baseline adherence to 10-week engagement and 10-week adherence. Pearson correlations were conducted to assess the relationship between baseline adherence and number of prescribed pills. All statistical analyses were performed using the R software, version 4.0.5 (The R Foundation).

## Results

**Figure 1:**
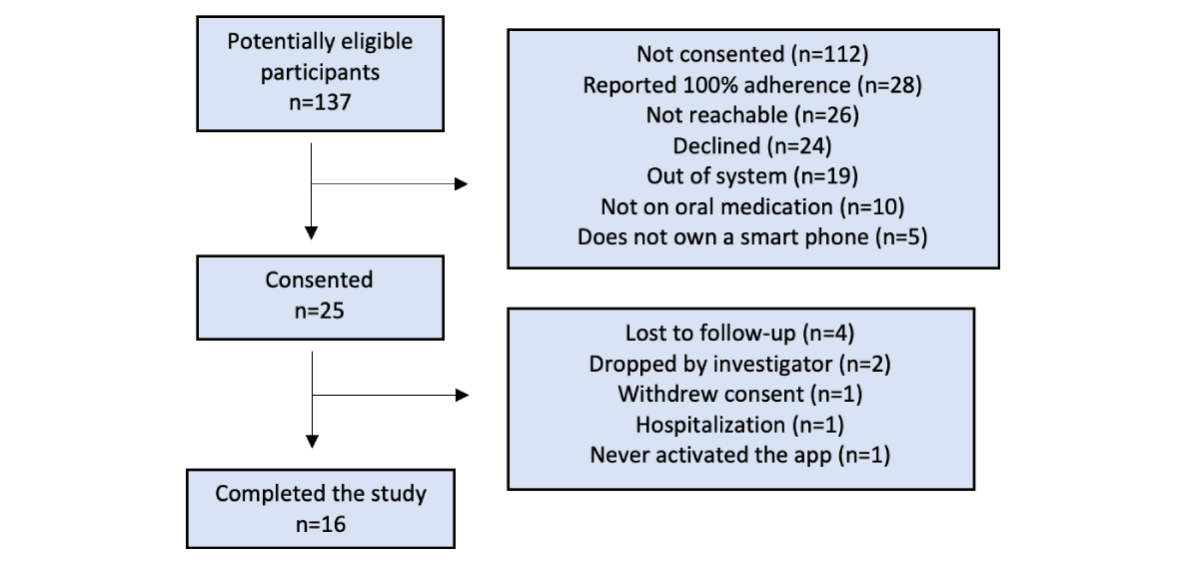
CONSORT (Consolidated Standards of Reporting Trials) flow diagram.

A total of 25 patients were enrolled in this pilot study between January and July 2020 (Figure 1); 72% (n=18) were female, and a majority were of a White racial background (n=12, 48%), followed by Black (n=6, 24%) and Asian (n=4, 16%). Median age was 24 years (Q1-Q3: 20.5-30). The participants were diagnosed with schizophrenia and related disorders (n=9, 36%), followed by major depressive disorder (n=8, 32%). Detailed characteristics of the patient sample are included in Table 1.

Treatment regime for each study participant is described in Multimedia Appendix 1. Study participants received treatment with a variety of oral antipsychotics, antidepressants, and mood stabilizers. The number of prescribed pills taken per day per subject varied from a minimum of 1 per day to a maximum of 9 per day (mean 2.82, SD 1.99). This measure did not correlate with baseline adherence (*r*=0.01).

**Figure 2:**
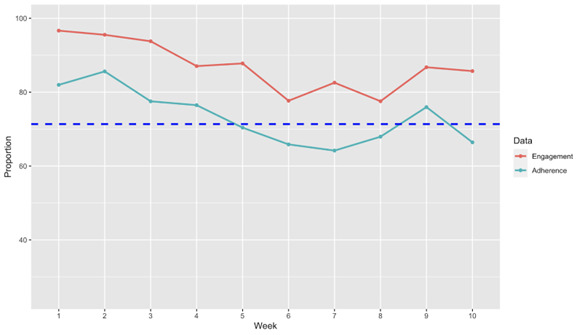
Summary measures of adherence and engagement throughout the 10-week study period. Blue dotted line represents mean baseline adherence of the sample.

Engagement and adherence were generally higher than baseline adherence but fluctuated throughout the study period (Figure 2). At study endpoint, engagement was higher compared to baseline (n=24, *Z*=–3.17; *P*<.001) but adherence was not different from baseline (n=24, *Z*=–0.59; *P*=.28). One study participant was removed from this analysis as the app was not downloaded. We conducted an additional sensitivity analysis including only those who finished participation (n=16), but the results remained unchanged for engagement (n=16, *Z*=–2.22; *P*=.01) and adherence (n=16, *Z*=–0.66; *P*=.25).

**Figure 3:**
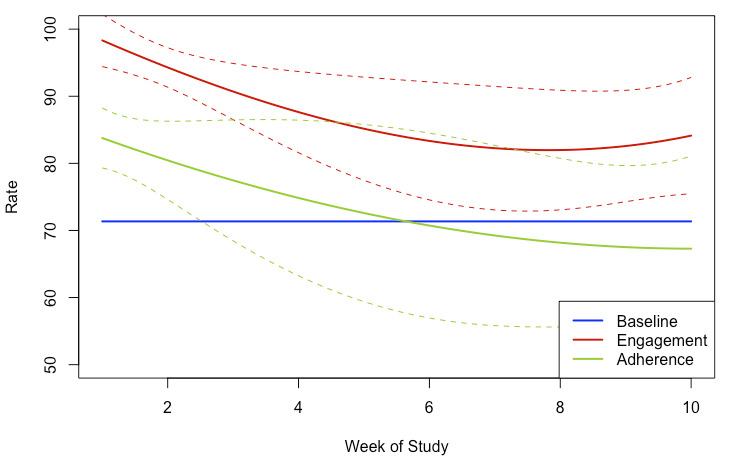
Time-varying effect model (TVEM) of engagement and adherence over time, plotting the estimated coefficient function for both engagement and adherence (solid line) with approximation of 95% confidence interval (dotted line) for the proportion at each time point (week).

We additionally conducted a TVEM to understand how engagement and adherence change over time, showing significantly higher engagement and adherence in the first 2 weeks compared with the last 8 weeks of the study (Figure 3).

**Table 1:** Sociodemographic characteristics of the patient sample (N=25).

Variable			Value
Age (years), mean (Q1-Q3)			24 (20.5-30)
**Sex, n (%)**			
	Female	18 (72)	
	Male	7 (28)	
**Race, n (%)**			
	White	12 (48)	
	Black or African American	6 (24)	
	Asian	4 (16)	
	Mixed or other	3 (12)	
**Primary diagnosis, n (%)**			
	Schizophrenia and related disorders	9 (36)	
	Major depressive disorder	8 (32)	
	Bipolar disorder	4 (16)	
	Schizoaffective disorder	2 (8)	
	Other	2 (8)	
**Marital status, n (%)**			
	Single	20 (80)	
	Married	4 (16)	
	Divorced or separated	1 (4)	
**Education status, n (%)**			
	Some college	11 (44)	
	High school	5 (20)	
	College graduate	3 (12)	
	Master’s degree	2 (8)	
	Some master’s	2 (8)	
	Unfinished high school	2 (8)	
**Employment status, n (%)**			
	Unemployed	15 (60)	
	Employed	10 (40)	
	Retired	0 (0)	
	Disabled	0 (0)	
**Insurance type, n (%)**			
	Private	16 (64)	
	Medicaid or Medicare	9 (36)	

## Discussion

In this pilot study, we show that financial incentives can be effectively delivered through an app in severe mental illness. We found that small financial incentives increased medication adherence during the initial 2 weeks of our follow-up period, yet this increase was not maintained at study endpoint.

Our findings add to previous studies showing great potential for behavioral economics-based financial incentives to impact patient outcomes in severe mental illness including medication adherence [24,25]; however, we additionally show that such incentives can be remotely delivered through an app, which allows real-time measures of engagement and adherence, thus paving the way for future studies to evaluate the efficacy different types of incentives or incentive combinations but also for clinicians to access daily adherence data, which could prompt specific interventions if nonadherence is detected.

Despite this study involving financial incentives, attrition was relatively high. This finding could relate to the amount of the incentive, lower than other recently published meta-analysis exploring strategies to incentivize medication adherence in the context of substance use disorders, reporting mean maximum daily earnings of over US $10 [38]. Alternatively, perhaps loss aversion strategies may be less effective in severe mental illness compared with traditional gain-framed incentives. Recent studies evaluating financial incentives to enhance adherence to oral treatment in depression show that escalating amounts up to US 7$ a day was more effective than de-escalating incentives or control groups [26]. Nonetheless, it is also possible that participants took some medications outside of their specific check-in window; therefore, pill count in each check-in photo may not truly capture all the medications an individual took that day, underestimating incentive effects. Lastly, specific app features could have influenced the results as well. Future study designs should include higher or escalating incentives, a larger sample size, an active control group, and additional measures of adherence.

The results should be interpreted with caution, as a pre-post single-arm study lacks control group and randomization, and factors unrelated to the intervention itself could be partially responsible for the differences detected. Nonetheless, this design can inform about the feasibility of offering financial incentives via an app to enhance medication adherence in severe mental illness. Engagement remained high during the initial 2 weeks of the study and was stable afterward, improving generally reported mental health app engagement rates [39]. Second, baseline measures of adherence were self-reported and thus subject to possible inaccuracies. However, chart and database reviews were conducted to confirm patient reports. Lastly, clinical outcomes were not measured, which is relevant as enhanced adherence may not necessarily reflect improved clinical outcomes [40].

In summary, financial incentives can be effectively delivered using an app. Leveraging behavioral economics and mobile health technology can increase medication adherence in the short term while maintaining high app engagement.

## Appendix

Multimedia Appendix 1 Supplementary Table 1.

## Abbreviations

TVEM: time-varying effect modeling

## References

[1] Sabaté E (2003). Adherence to Long-Term Therapies: Evidence for Action.

[2] Dell'Osso B, Albert U, Carrà G, Pompili M, Nanni MG, Pasquini M, Poloni N, Raballo A, Sambataro F, Serafini G, Viganò C, Demyttenaere K, McIntyre RS, Fiorillo A (2020). How to improve adherence to antidepressant treatments in patients with major depression: a psychoeducational consensus checklist. Ann Gen Psychiatry.

[3] Leclerc E, Mansur RB, Brietzke E (2013). Determinants of adherence to treatment in bipolar disorder: a comprehensive review. J Affect Disord.

[4] Sendt K, Tracy DK, Bhattacharyya S (2015). A systematic review of factors influencing adherence to antipsychotic medication in schizophrenia-spectrum disorders. Psychiatry Res.

[5] Leucht S, Tardy M, Komossa K, Heres S, Kissling W, Salanti G, Davis JM (2012). Antipsychotic drugs versus placebo for relapse prevention in schizophrenia: a systematic review and meta-analysis. The Lancet.

[6] Furukawa TA, Levine SZ, Tanaka S, Goldberg Y, Samara M, Davis JM, Cipriani A, Leucht S (2015). Initial severity of schizophrenia and efficacy of antipsychotics: participant-level meta-analysis of 6 placebo-controlled studies. JAMA Psychiatry.

[7] Park LT, Zarate CA (2019). Depression in the Primary Care Setting. N Engl J Med.

[8] Fournier JC, DeRubeis RJ, Hollon SD, Dimidjian S, Amsterdam JD, Shelton RC, Fawcett J (2010). Antidepressant drug effects and depression severity: a patient-level meta-analysis. JAMA.

[9] Krivoy A, Balicer RD, Feldman B, Hoshen M, Zalsman G, Weizman A, Shoval G (2016). Adherence to Antidepressants Is Associated With Lower Mortality. J. Clin. Psychiatry.

[10] Cramer JA, Rosenheck R (1998). Compliance with medication regimens for mental and physical disorders. Psychiatr Serv.

[11] Lacro JP, Dunn LB, Dolder CR, Leckband SG, Jeste DV (2002). Prevalence of and risk factors for medication nonadherence in patients with schizophrenia: a comprehensive review of recent literature. J Clin Psychiatry.

[12] Lingam R, Scott J (2002). Treatment non-adherence in affective disorders. Acta Psychiatr Scand.

[13] Sajatovic M, Valenstein M, Blow FC, Ganoczy D, Ignacio RV (2006). Treatment adherence with antipsychotic medications in bipolar disorder. Bipolar Disord.

[14] Sawada N, Uchida H, Suzuki T, Watanabe K, Kikuchi T, Handa T, Kashima H (2009). Persistence and compliance to antidepressant treatment in patients with depression: A chart review. BMC Psychiatry.

[15] Sheehan DV, Keene MS, Eaddy M, Krulewicz S, Kraus JE, Carpenter DJ (2008). Differences in medication adherence and healthcare resource utilization patterns: older versus newer antidepressant agents in patients with depression and/or anxiety disorders. CNS Drugs.

[16] Keyloun KR, Hansen RN, Hepp Z, Gillard P, Thase ME, Devine EB (2017). Adherence and Persistence Across Antidepressant Therapeutic Classes: A Retrospective Claims Analysis Among Insured US Patients with Major Depressive Disorder (MDD). CNS Drugs.

[17] Robinson D, Woerner MG, Alvir JMJ, Bilder R, Goldman R, Geisler S, Koreen A, Sheitman B, Chakos M, Mayerhoff D, Lieberman JA (1999). Predictors of relapse following response from a first episode of schizophrenia or schizoaffective disorder. Arch Gen Psychiatry.

[18] Ascher-Svanum H, Faries DE, Zhu B, Ernst FR, Swartz MS, Swanson JW (2006). Medication adherence and long-term functional outcomes in the treatment of schizophrenia in usual care. J Clin Psychiatry.

[19] Law MR, Soumerai SB, Ross-Degnan D, Adams AS (2008). A longitudinal study of medication nonadherence and hospitalization risk in schizophrenia. J Clin Psychiatry.

[20] Novick D, Haro JM, Suarez D, Perez V, Dittmann RW, Haddad PM (2010). Predictors and clinical consequences of non-adherence with antipsychotic medication in the outpatient treatment of schizophrenia. Psychiatry Res.

[21] Hong J, Reed C, Novick D, Haro JM, Aguado J (2011). Clinical and economic consequences of medication non-adherence in the treatment of patients with a manic/mixed episode of bipolar disorder: results from the European Mania in Bipolar Longitudinal Evaluation of Medication (EMBLEM) study. Psychiatry Res.

[22] Akerblad A-C, Bengtsson Finn, von Knorring Lars, Ekselius Lisa (2006). Response, remission and relapse in relation to adherence in primary care treatment of depression: a 2-year outcome study. Int Clin Psychopharmacol.

[23] Weiden PJ, Kozma C, Grogg A, Locklear J (2004). Partial compliance and risk of rehospitalization among California Medicaid patients with schizophrenia. Psychiatr Serv.

[24] Guinart D, Kane JM (2019). Use of Behavioral Economics to Improve Medication Adherence in Severe Mental Illness. Psychiatr Serv.

[25] Guinart D, Kane JM (2020). Incentivizing Is Not Coercing: A Commentary. Psychiatr Serv.

[26] Marcus SC, Reilly ME, Zentgraf K, Volpp KG, Olfson M (2021). Effect of Escalating and Deescalating Financial Incentives vs Usual Care to Improve Antidepressant Adherence: A Pilot Randomized Clinical Trial. JAMA Psychiatry.

[27] Priebe S, Yeeles K, Bremner S, Lauber C, Eldridge S, Ashby D, David AS, O'Connell N, Forrest A, Burns T (2013). Effectiveness of financial incentives to improve adherence to maintenance treatment with antipsychotics: cluster randomised controlled trial. BMJ.

[28] Noordraven EL, Wierdsma AI, Blanken P, Bloemendaal AFT, Staring ABP, Mulder CL (2017). Financial incentives for improving adherence to maintenance treatment in patients with psychotic disorders (Money for Medication): a multicentre, open-label, randomised controlled trial. Lancet Psychiatry.

[29] Wellth Solution. Wellth.

[30] Ahmed I, Ahmad NS, Ali S, Ali S, George A, Saleem Danish H, Uppal E, Soo J, Mobasheri MH, King D, Cox B, Darzi A (2018). Medication Adherence Apps: Review and Content Analysis. JMIR Mhealth Uhealth.

[31] Sobolev M (2021). Digital Nudging: Using Technology to Nudge for Good. SSRN Journal.

[32] World Medical Association (2013). World Medical Association Declaration of Helsinki: ethical principles for medical research involving human subjects. JAMA.

[33] Patel MS, Asch DA, Rosin Roy, Small DS, Bellamy SL, Heuer J, Sproat S, Hyson C, Haff N, Lee SM, Wesby L, Hoffer K, Shuttleworth D, Taylor DH, Hilbert V, Zhu J, Yang L, Wang X, Volpp KG (2016). Framing Financial Incentives to Increase Physical Activity Among Overweight and Obese Adults: A Randomized, Controlled Trial. Ann Intern Med.

[34] Halpern SD, French B, Small DS, Saulsgiver K, Harhay MO, Audrain-McGovern J, Loewenstein G, Brennan TA, Asch DA, Volpp KG (2015). Randomized Trial of Four Financial-Incentive Programs for Smoking Cessation. N Engl J Med.

[35] Matthews JN, Altman DG, Campbell MJ, Royston P (1990). Analysis of serial measurements in medical research. BMJ.

[36] Tan X, Shiyko MP, Li R, Li Y, Dierker L (2012). A time-varying effect model for intensive longitudinal data. Psychol Methods.

[37] Dziak J, Coffman DL, Li R, Litson K, Chakraborti Y Package ‘tvem’. R Project.

[38] Bolívar HA, Klemperer EM, Coleman SRM, DeSarno M, Skelly JM, Higgins ST (2021). Contingency Management for Patients Receiving Medication for Opioid Use Disorder: A Systematic Review and Meta-analysis. JAMA Psychiatry.

[39] Baumel A, Muench F, Edan S, Kane JM (2019). Objective User Engagement With Mental Health Apps: Systematic Search and Panel-Based Usage Analysis. J Med Internet Res.

[40] Barankay I, Reese PP, Putt ME, Russell LB, Loewenstein G, Pagnotti D, Yan J, Zhu J, McGilloway R, Brennan T, Finnerty D, Hoffer K, Chadha S, Volpp KG (2020). Effect of Patient Financial Incentives on Statin Adherence and Lipid Control: A Randomized Clinical Trial. JAMA Netw Open.

