# Supplementary material for: A Digital Intervention Using Daily Financial Incentives to Increase Medication Adherence in Severe Mental Illness: Single-Arm Longitudinal Pilot Study
Source: JMIR Ment Health. 2022 Oct 12;9(10):e37184. doi: 10.2196/37184 (PMC9607890; doi:10.2196/37184)
Supplement: Multimedia Appendix 1 [file mental_v9i10e37184_app1.docx]

**Supplementary Table 1.** Treatment regime of study participants

| Treatment | Dose  (Total daily dose) | Regime | Duration (months) |
| --- | --- | --- | --- |
| Subject 1 |  |  |  |
| Trazodone  Escitalopram  Dextroamphetamine  Clonidine | 100 mg  20 mg  25 mg  0.4 mg | q.d.  q.d.  q.d.  q.d. | 30  23  251  71 |
| Subject 2 |  |  |  |
| Aripiprazole  Sertraline  Bupropion | 10 mg  200 mg  300 mg | q.d.  q.d.  q.d. | 24  24  24 |
| Subject 3 |  |  |  |
| Aripiprazole  Lithium | 5 mg  450 mg | q.d.  q.d. | 10  10 |
| Subject 4 |  |  |  |
| Bupropion  Venlafaxine | 450 mg  225 mg | q.d.  q.d. | 5  5 |
| Subject 5 |  |  |  |
| Olanzapine  Aripiprazole  Escitalopram  Benztropine | 15 mg  15 mg  20 mg  1 mg | q.d.  q.d.  q.d.  q.d. | 5  2  2  3 |
| Subject 6 |  |  |  |
| Bupropion  Lamotrigine | 300 mg  200 mg | q.d.  q.d. | 19  64 |
| Subject 7 |  |  |  |
| Aripiprazole  Lithium | 5 mg  900 mg | q.d.  q.d. | 2  2 |
| Subject 8 |  |  |  |
| Aripiprazole  Sertraline  Trazodone | 5 mg  100 mg  50 mg | q.d.  q.d.  q.d. | 4  4  1 |
| Subject 9 |  |  |  |
| Olanzapine  Escitalopram | 10 mg  20 mg | q.d.  q.d. | 14  14 |
| Subject 10 |  |  |  |
| Sertraline | 75 mg | q.d. | 1 |
| Subject 11 |  |  |  |
| Olanzapine  Citalopram | 2.5 mg  10 mg | q.d.  q.d. | 240  240 |
| Subject 12 |  |  |  |
| Sertraline | 125 mg | q.d. | 11 |
| Subject 13 |  |  |  |
| Aripiprazole | 5 mg | q.d. | 1 |
| Subject 14 |  |  |  |
| Risperidone  Fluoxetine | 0.75 mg  60 mg | b.i.d.  q.d. | 8  8 |
| Subject 15 |  |  |  |
| Sertraline | 50 mg | q.d. | 3 |
| Subject 16 |  |  |  |
| Divalproex | 1500 mg | q.d. | 120 |
| Subject 17 |  |  |  |
| Aripiprazole  Lithium  Divalproex  Clonazepam | 5 mg  900 mg  1250 mg  1 mg | q.d.  q.d.  q.d.  q.d. | 4  24  12  60 |
| Subject 18 |  |  |  |
| Quetiapine | 50 mg | q.d. | 1 |
| Subject 19 |  |  |  |
| Risperidone | 4 mg | q.d. | 8 |
| Subject 20 |  |  |  |
| Risperidone  Benztropine | 4 mg  1 mg | q.d.  q.d. | 3  3 |
| Subject 21 |  |  |  |
| Aripiprazole  Trazodone  Benztropine | 15 mg  50 mg  0.5 mg | q.d.  q.d.  q.d. | 1  1  1 |
| Subject 22 |  |  |  |
| Risperidone  Benztropine | 3 mg  1 mg | q.d.  q.d. | 1  1 |
| Subject 23 |  |  |  |
| Mirtazapine | 30 mg | q.d. | 2 |
| Subject 24 |  |  |  |
| Olanzapine | 10 mg | q.d. | 3 |
| Subject 25 |  |  |  |
| Risperidone  Lorazepam  Benztropine | 2 mg  1 mg  2 mg | q.d.  q.d.  q.d. | 30  30  30 |

Abbreviations: q.d=once a day, b.i.d.=twice a day
